# Role for the *Plasmodium* sporozoite-specific transmembrane protein S6 in parasite motility and efficient malaria transmission

**DOI:** 10.1111/j.1462-5822.2008.01252.x

**Published:** 2008-10-31

**Authors:** Marion Steinbuechel, Kai Matuschewski

**Affiliations:** 1 Department of Parasitology, Heidelberg University School of Medicine69120 Heidelberg, Germany; 2 Parasitology Unit, Max Planck Institute for Infection Biology10117 Berlin, Germany

## Abstract

Malaria transmission occurs by intradermal deposition of *Plasmodium* sporozoites during the infectious bite of a female *Anopheles* mosquito. After formation in midgut-associated oocysts sporozoites actively enter mosquito salivary glands and subsequently invade host hepatocytes where they transform into clinically silent liver stages. To date, two sporozoite-specific transmembrane proteins have been identified that perform vital functions in natural malaria transmission. The sporozoite invasin TRAP drives sporozoite motility and target cell entry whereas the adhesin MAEBL mediates sporozoite recognition of and attachment to salivary glands. Here, we demonstrate that the sporozoite-specific transmembrane protein S6 is required for efficient malaria transmission to the vertebrate host. Targeted deletion of S6 results in severe impairment of sporozoite gliding motility and invasion of mosquito salivary glands. During sporozoite maturation S6 expression is tightly regulated by transcriptional and translational control. We propose that S6 functions together with TRAP/MIC2 family invasins to direct fast, efficient and specific cell entry and, ultimately, life cycle progression of the malaria sporozoite.

## Introduction

Malaria remains the most important vector-borne infectious disease worldwide. It is caused by unicellular *Plasmodium* parasites that have the exceptional capacity to invade and develop within host erythrocytes. Malarial parasites are transmitted during the bloodmeal of an infected female *Anopheles* mosquito (Vanderberg and Frevert, 2004; Amino *et al*., 2006; Yamauchi *et al*., 2007). The contagious *Plasmodium* forms, sporozoites, are highly motile and actively enter the blood circulation in order to reach the liver where they undergo a dramatic transition and expansion phase. This pre-erythrocytic schizogony is clinically silent and results in the generation of thousands of pathogenic merozoites from a single sporozoite (Prudêncio *et al*., 2006). *Plasmodium* liver stage development compensates for the low numbers of transmitted sporozoites, a major bottleneck of the *Plasmodium* life cycle. Therefore, understanding the cellular and molecular mechanisms of sporozoite maturation, motility and invasion into host cells may assist in developing new potent intervention strategies against malaria.

*Plasmodium* sporozoites are formed inside oocysts, in a process termed sporogony, and share the unifying features of all apicomplexan invasive stages, i.e. they contain secretory organelles and display active locomotion (Sinden and Matuschewski, 2005). Sporozoites are covered with a dense coat made of circumsporozoite protein (CSP), the major surface coat protein (Nardin *et al*., 1982). Once mature, sporozoites become motile and egress from oocysts into the haemolymph (Aly and Matuschewski, 2005; Wang *et al*., 2005). Upon contact with their final target organ in the mosquito vector, the salivary glands, they specifically bind to and penetrate the distal portion of the lateral lobes resulting in accumulation of mature, infectious sporozoites in the salivary duct and potential transmission to the mammalian host.

The sporozoite-specific transmembrane surface protein TRAP (thrombospondin-related anonymous protein) is the founding member of a protein family that mediates cell invasion in Apicomplexan parasites (Tomley and Soldati, 2001). *TRAP* deficiency or mutations in key cytoplasmic and extracellular amino acid residues result in ablation of sporozoite locomotion and host cell entry (Sultan *et al*., 1997; Kappe *et al*., 1999; Wengelnik *et al*., 1999; Matuschewski *et al*., 2002a). The unifying structural features of TRAP/MIC2 family invasins are combinations of extracellular adhesive modules, i.e. the von Willebrand factor A-domain (A-domain) and the thrombospondin type I repeat (TSR), a cleavable transmembrane domain, and a cytoplasmic tail domain (CTD) that contains a penultimate tryptophan residue preceded by multiple negatively charged amino acids. According to the present model an extracellular binding event is transmitted to the cytoplasmic domain that links the transmembrane protein to the actin/myosin motor of the sporozoite (Keeley and Soldati, 2004; Schüler and Matuschewski, 2006). Up to date, TRAP remains the only known sporozoite-specific TRAP/MIC2 family protein that performs an essential role for locomotion and life cycle progression of the malaria sporozoite (Sultan *et al*., 1997; Matuschewski, 2006). Another sporozoite transmembrane protein, termed apical membrane antigen/erythrocyte binding-like protein (MAEBL), mediates salivary gland recognition and adhesion, but is dispensable for gliding locomotion (Kariu *et al*., 2002; Preiser *et al*., 2004; Fu *et al*., 2005; Sáenz *et al*., 2008).

In this study we characterized the *in vivo* function of a sporozoite-specific transmembrane protein, S6, which was initially identified in a screen for sporozoite-enriched transcripts (Kaiser *et al*., 2004). We show that *S6* is important for efficient sporozoite locomotion and target cell entry. Apparently, *Plasmodium* sporozoites employ at least three stage-specific transmembrane proteins to guarantee efficient transmission to the mammalian host.

## Results

### *S6* is specifically expressed in *Plasmodium* sporozoites

**Fig. 1:**
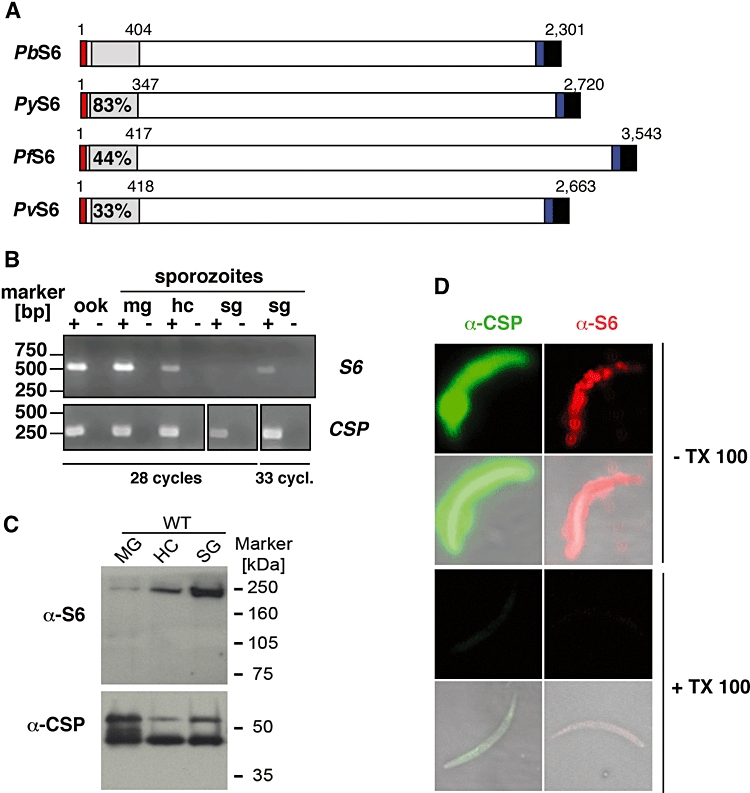
Expression of *Plasmodium berghei S6* during sporozoite maturation. A. Schematic diagram of the primary structure of *Plasmodium* S6 proteins. The predicted signal peptide, the carboxy-terminal transmembrane span and cytoplasmic tail domain of TRAP family invasins are boxed in red, blue and black respectively. Amino acid sequence identities of the amino-terminal region (grey box) of the *P. yoelii* (PY04986, re-annotated), *P. falciparum* (PF14_0404), *P. vivax* (Pv118360, re-annotated) S6 orthologues are indicated as percentage of identical residues compared with the *P. berghei* (FJ160771) sequence. B. Downregulation of *PbS6* transcripts during sporozoite maturation. Shown is a RT-PCR analysis of *PbS6* mRNA in ookinetes (ook), and midgut (mg)-, haemocoel (hc)- and salivary gland (sg)-associated sporozoites. The abundant *PbCSP* transcript was added as a control. *PbS6* and *PbCSP* were amplified at 28 and 24 PCR cycles respectively. Note that the *S6* signal in salivary gland sporozoites can only be revealed by amplification at higher cycle number (33 cycles). C. Western blot analysis of PbS6 during sporozoite maturation. Sporozoite extracts from 100 000 wild-type oocyst, haemocoel or salivary gland sporozoites were separated on an 8% SDS-PAGE and probed with a polyclonal anti-*Pb*S6 antiserum or the CSP monoclonal antibody as a loading control, followed by peroxidase-coupled anti-rabbit or anti-mouse antisera. D. Immunolocalization of *Pb*S6 in sporozoites. Fixed sporozoites were incubated with a monoclonal anti-CSP antibody (green) and the polyclonal anti-*Pb*S6 antiserum (red) and their corresponding fluorescently labelled secondary antibodies. Note that solubilization of the plasma membrane with Triton X-100 (TX 100) abolishes the strong plasma membrane labelling of both antibodies.

*S6* (PF14_0404) was first discovered in a screen designed to identify *Plasmodium yoelii* sporozoite-specific genes that are absent in blood stages (Kaiser *et al*., 2004). The orthologous *Plasmodium berghei S6* gene was identified in the genome database and encodes a protein of 2301 amino acid residues (Fig. 1A). The S6 protein appears to be a surface-exposed type I transmembrane protein and exhibits two main remarkable features. (i) It contains a carboxy-terminal TRAP family-like CTD, including the penultimate tryptophan and a cluster of negatively charged residues (Kaiser *et al*., 2004). These residues within the CTD are a hallmark of TRAP family invasins and play crucial roles during gliding locomotion (Kappe *et al*., 1999; Heiss *et al*., 2008). (ii) The large extracellular portion consists largely of low-complexity regions and lacks apparent cell adhesion modules, such as TSRs and A-domains.

We first analysed *S6* transcript abundance during sporozoite maturation (Fig. 1B). cDNAs generated from ookinetes, oocyst, haemocoel and salivary gland-associated sporozoites were used as templates for semi-quantitative RT-PCR. The transcript of the major sporozoite surface protein *CSP* served for data standardization. *PbS6* expression was the highest in early mosquito stages, including ookinetes and young oocyst sporozoites. *S6* was detectable in haemocoel sporozoites, but transcript levels were low in salivary gland-associated sporozoites. As observed previously (Matuschewski *et al*., 2002b), *CSP* transcription is slightly downregulated in mature salivary gland-associated sporozoites. In marked contrast, *S6* can only be detected at higher cycle numbers, suggesting only residual transcript levels in mature sporozoites. In support of our findings, we independently isolated *S6* as one of the most abundant transcript in a suppression subtractive hybridization screen to select for genes that are downregulated during sporozoite maturation (our unpublished data). Therefore, transcriptional control of *S6* expression is markedly different from *CSP* and *TRAP* (Matuschewski *et al*., 2002b)*.* Collectively, our data suggest that *S6* transcription is downregulated during sporozoite maturation.

### *S6* expression is translationally controlled

We next wanted to examine protein expression during sporozoite formation and raised polyclonal antisera against *Pb*S6. We first analysed protein expression during sporozoite maturation by Western blot analysis (Fig. 1C). Using the *Pb*S6 antiserum we detected a specific signal at the expected size of ∼260 kDa. Unexpectedly, the S6 protein displays an increasing accumulation from oocyst to salivary gland sporozoites, differing substantially from the transcriptional profile, suggesting that S6 protein synthesis is delayed compared with gene transcription. This finding is reminiscent of translational repression, which has been previously observed for gametocyte-specific genes that are repressed by a DDX6 family member of DEAD-box RNA helicases, termed development of zygote inhibited (DOZI) (Mair *et al*., 2006).

To confirm the immunoblot analysis and detect the localization of *Pb*S6 we next studied wild-type (WT) sporozoites by immunofluorescence microscopy. In fixed haemocoel sporozoites we detected a punctuate pattern for PbS6 that contrasted with the uniform distribution of CSP (Fig. 1D). In order to confirm the surface localization of S6 we treated sporozoites with the detergent Triton X-100 to remove the plasma membrane. Reactivity with either CSP or S6 antisera was ablated in detergent-treated sporozoites suggesting a comparable localization. Together our findings indicate that the S6 protein localizes to the sporozoite plasma membrane and its expression is under stage-specific transcriptional and translational control.

### Generation of *s6(−)* parasites

**Fig. 2:**
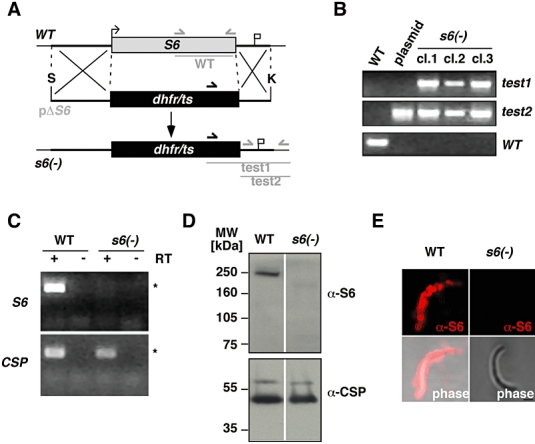
Targeted gene disruption of *P. berghei S6*. A. Replacement strategy to generate *s6*(*−)* parasite lines. The wild-type (WT) *S6* genomic locus was targeted with a SacII/KpnI-linearized replacement plasmid (pΔS6) containing 5′ and 3′ untranslated regions of the *PbS6* open reading frame and the positive selectable marker, i.e. *Toxoplasma gondii* dihydrofolate reductase/thymidylate synthase (*dhfr/ts*). Upon a double-cross-over event, the endogenous *S6* locus is replaced by the selection marker resulting in a loss-of-function parasite line, i.e. a replacement line, *s6*(*−)*, in the green fluorescent *Pb*ANKA strain (Janse *et al*., 2006). Replacement-specific test primer combinations and corresponding fragments are indicated by arrows and grey lines respectively. B. Replacement-specific PCR analysis of the *s6*(*−)* parasite lines. The predicted gene targeting is confirmed by primer combination ‘test1’, and presence of the targeting construct by combination ‘test2’. The WT-specific test PCR confirms the absence of residual WT parasites in three representative clonal *s6*(*−)* lines. C. Depletion of *S6* transcripts in *s6*(*−)* parasites. cDNAs from WT and *s6*(*−)* midgut-associated sporozoites were amplified with *S6*-specific primers and *CSP* primers as controls. D. Absence of S6 protein in *s6*(*−)* parasites. Shown is a Western blot analysis with the polyclonal anti-*Pb*S6 antiserum and the CSP monoclonal antibody as a loading control. E. Ablation of S6 plasma membrane staining in *s6*(*−)* parasites. Fixed WT and *s6*(*−)* haemocoel sporozoites were incubated with the polyclonal anti-*Pb*S6 antiserum (red) and their corresponding fluorescently labelled secondary antibody.

The tight expression regulation and spatial distribution of S6 is indicative of an important cellular function that is likely restricted to sporozoites in the mosquito vector. To identify the *in vivo* roles of *S6* in *P. berghei* life cycle progression we generated a *Pbs6(−)* parasite line by allelic exchange (Fig. 2A). Importantly, we could select for viable blood-stage parasites that contain a targeted deletion of the *S6* open reading frame, in good agreement with sporozoite-specific gene expression and absence of transcripts in blood stages (Kaiser *et al*., 2004). The parental parasite populations were subcloned to generate clonal parasite lines, termed *s6(−)* (Fig. 2A).

Successful gene replacement in the *s6(−)* clones was confirmed by integration-specific PCR (Fig. 2B) and absence of *S6* transcripts by RT-PCR analysis (Fig. 2C). Western blot analysis (Fig. 2D) confirmed successful *S6* depletion by the replacement strategy and the specificity of the anti-S6 antiserum. In good agreement, *s6(−)* haemocoel sporozoites displayed no detectable immunofluorescence staining when incubated with the S6 antiserum (Fig. 2E). In conclusion, successful generation of *S6* loss-of-function parasites demonstrated that this gene is dispensable for propagation of asexual blood-stage parasites.

### Impairment of mosquito salivary gland invasion in *s6(−)* sporozoites

**Fig. 3:**
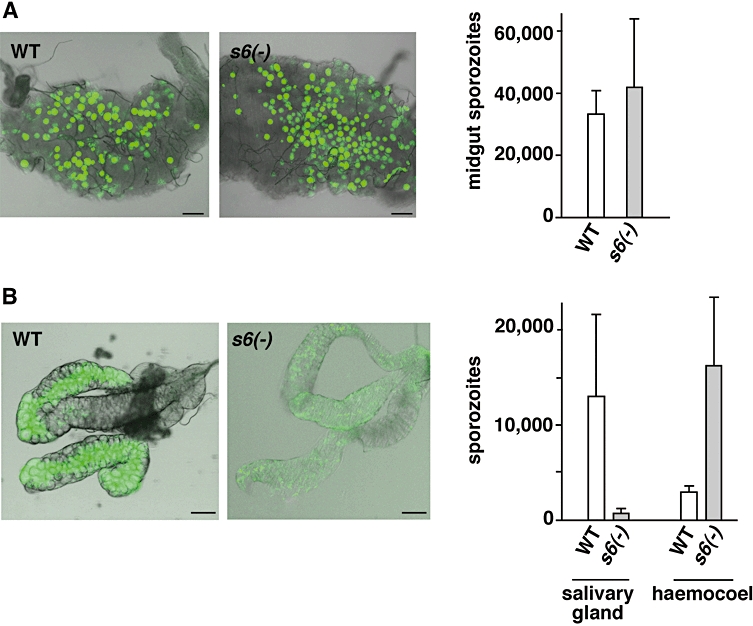
*S6(−)* sporozoites are defective in mosquito salivary gland invasion. A. WT and *s6*(*−)* parasites produce similar numbers of oocysts (left) and midgut-associated sporozoites (right). Oocysts were counted based on their GFP fluorescence and sporozoite numbers calculated per infected mosquito from at least three independent feeding experiments each. B. Deficiency in salivary gland invasion in *S6* loss-of-function mutants. Shown are representative fixed salivary glands after infection (left) and quantifications of salivary gland-associated sporozoites per infected mosquito (right). Note the accumulation of haemocoel sporozoites in the *s6*(*−)* mutants, as shown by quantifications of WT and *s6*(*−)* haemocoel sporozoites.

We next examined the fate of the *s6(−)* parasite lines during *Plasmodium* life cycle progression. Gametocyte formation, exflagellation, transmission to mosquitoes, as well as midgut infectivity (oocyst development and morphology) of *s6(−)* parasites were normal when compared with WT (data not shown). In general, oocyst development is complete at day 14 after mosquito infection. At this stage, *s6(−)* parasites were indistinguishable from WT parasites (Fig. 3A). Quantification of midgut-associated sporozoites revealed no differences between the two parasite lines. In marked contrast, *s6(−)* sporozoites were severely impaired in salivary gland invasion (Fig. 3B). The quantification of isolated salivary gland sporozoites showed a dramatic decrease in *s6(−)* parasite numbers when compared with WT. Importantly, the observed deficiency to enter mosquito salivary glands was accompanied by substantial accumulation of viable sporozoites in the mosquito haemocoel (Fig. 3C). These findings indicate that *S6* is not involved in sporozoite egress from oocysts but plays an important role prior to transmission to the mammalian host.

### Deficient motility in *s6(−)* sporozoites

**Fig. 4:**
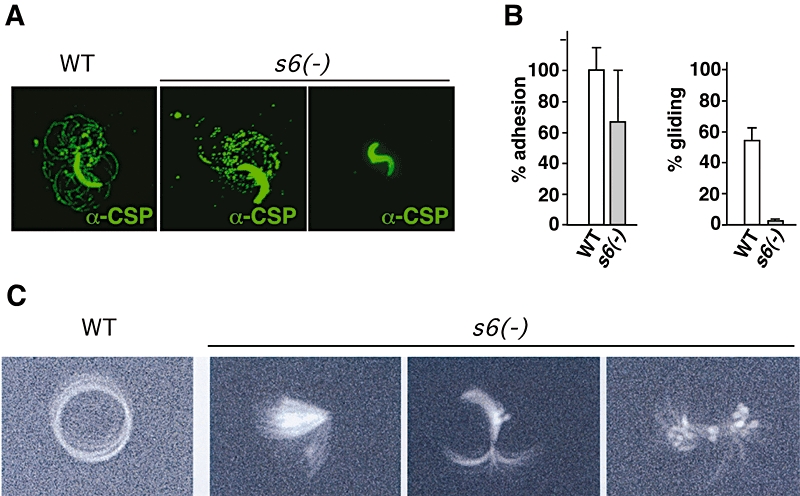
*S6(−)* sporozoites are impaired in gliding locomotion. A. Representative immunofluorescence images of WT and *s6*(*−)* sporozoites, revealed with the anti-CSP antibody. WT sporozoites (left) display the typical circular continuous gliding pattern, whereas *s6*(*−)* sporozoites either are non-motile (right) or display discontinuous circular gliding (centre). B. Quantification of sporozoite adhesion and gliding trails by indirect immunofluorescence microscopy. A total of 10 000 haemocoel sporozoites of either WT or *s6*(*−)* parasites were deposited onto BSA-coated coverslips and incubated for 15 min at 37C. Trails were visualized with the anti-CSP monoclonal antibody. Shown is the mean percentage (±SD) of adherent sporozoites (shown as percentage of WT adhesion) and gliding sporozoites (trails ≥ 1 circle) from three independent experiments. C. Overlays of time-lapse micrographs of WT and *s6*(*−)* sporozoites. Shown are combined sequential images (3 s intervals) of gliding WT and *s6*(*−)* sporozoites. *S6*(*−)* sporozoites display either (i) attached waiving, (ii) incomplete circular motility or (iii) irregular movement. Note that only substrate-dependent productive movement is impaired, while attachment, bending and flexing are maintained.

The observed deficiency in salivary gland invasion resembles the phenotype of *trap(−)* sporozoites, which lost their ability to invade target cells and perform active gliding locomotion (Sultan *et al*., 1997). We therefore compared gliding motility of mutant and WT sporozoites. Because *s6(−)* sporozoites are severely impaired in salivary gland invasion, we tested motility of sporozoites isolated from the mosquito haemocoel by indirect immunofluorescence staining of CSP deposited into the trails of gliding sporozoites (Fig. 4A). Only rarely did we detect sporozoites with trails, which displayed a more discontinuous pattern when compared with productive motility of WT sporozoites. A quantitative analysis of gliding locomotion revealed that on average 55% of adherent WT haemocoel sporozoites showed a trail of one circle or greater (Fig. 4B). In marked contrast, only very few sporozoites (3%) displayed trail patterns that were always discontinuous.

The vast majority of *s6(−)* sporozoites were active and displayed various patterns of non-productive motility, such as attached waving, bending and flexing, and pendulum-like movements (see also Movie S1). To confirm these findings we analysed sporozoite motility by phase-contrast microscopy (Fig. 4C). This analysis confirmed that sporozoite adhesion *in vitro* occurs normally in the absence of *S6*. *S6(−)* haemocoel sporozoites adhere with one end and display the immature and non-productive motility patterns, but apparently lost their ability to glide over long distances (Fig. 4C). Together, our findings demonstrate a crucial role for *S6* in sporozoite locomotion.

### *S6(−)* sporozoites are impaired in transmission to the mammalian host

**Fig. 5:**
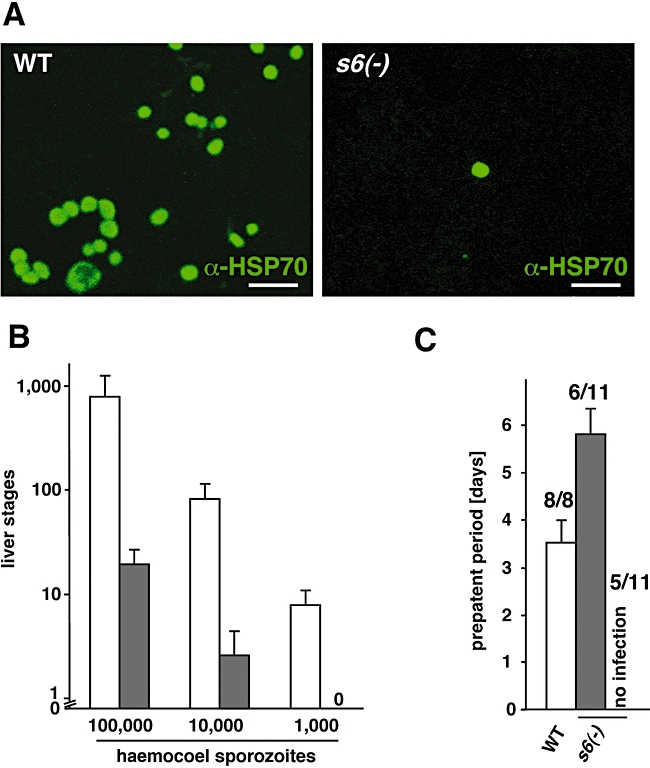
*S6(−)* sporozoites are defective in infectivity to the mammalian host. A. Representative immunofluorescence images of WT and *s6*(*−)* liver stages, 48 h after hepatocyte invasion. Infected hepatoma cells were fixed, permeabilized and stained with monoclonal anti-*Pb*HSP70 antibody. B. Quantification of parasite liver stages. Haemocoel sporozoites of either WT or *s6*(*−)* parasites were added in the numbers indicated to subconfluent hepatoma cells and incubated for 48 h. Shown are total liver stages from three independent experiments (mean value ± SD). Note that WT and mutant parasites differ by at least one order of magnitude (logarithmic scale). C. Infectivity of WT and *s6*(*−)* parasites by natural malaria transmission. Highly susceptible young Sprague/Dawley rats were exposed to five infected mosquitoes. The prepatent period was determined by daily examination of Giemsa-stained blood smears (mean value ± SD). Note that a substantial portion of animals develops malaria, despite very low salivary gland infection rates.

We finally tested whether the observed defects in gliding locomotion translate into a complete block of transmission to the mammalian host. In order to quantify the invasion capacity of *s6(−)* sporozoites we isolated haemocoel sporozoites by gentle perfusion of infected mosquitoes. When these sporozoites were added to subconfluent hepatoma cells and stained for mature liver stages at 48 h after sporozoite infection we consistently detected mature liver stages in *s6(−)*-infected hepatoma cells, albeit at greatly reduced numbers (Fig. 5A). Our quantitative analysis revealed a consistent reduction in liver stage numbers by at least an order of magnitude *in vitro* (Fig. 5B).

To exclude multiple roles of *S6* during hepatocyte invasion and liver stage development, we incubated haemocoel sporozoites with subconfluent hepatoma cells for 90 min and quantified intra- and extracellular sporozoites. While 18% of WT sporozoites productively invaded, only 0.5% of *s6(−)* sporozoites were located inside hepatoma cells (Table 1), suggesting that *S6* functions exclusively during sporozoite entry and not in subsequent steps of liver stage maturation.

**Table 1:** Infectivity of *s6(−)* haemocoel sporozoites.

Parasite population	Invasion^a^	No. injected sporozoites^b^	No. infected/No. injected^c^	Prepatent period (days)^d^
WT	18%	1 000	10/10	4.7
		10 000	8/8	4.6
		100 000	3/3	3.3
*s6(−)*	0.5%	1 000	1/10	(5.0)
		10 000	8/8	5.1
		100 000	5/5	3.4

a Invasion was assessed by differential immunostaining of extra- and intracellular sporozoites.

b Haemocoel sporozoites were injected intravenously at the numbers indicated.

c Highly susceptible Sprague/Dawley rats were infected.

d The prepatent period is the time until detection of the first blood stage by daily examination of Giemsa-stained blood smears.

When we tested natural malaria transmission through by-bite feedings with five infected *s6(−)* mosquitoes on naïve, young Sprague/Dawley rats, which are highly susceptible for *P. berghei* sporozoite infection, we detected a substantial proportion of successful, albeit delayed, blood-stage infections (Fig. 5C). This result confirms that a small proportion of *s6(−)* sporozoites retains the capacity to invade the salivary glands and is able to complete the entire *Plasmodium* life cycle. As expected, the observed defect in sporozoite locomotion and invasion to mosquito salivary glands translates into a substantial delay or absence of infection during natural transmission. To confirm that the main role of *S6* is during sporozoite entry of salivary glands, we bypassed the life cycle and injected haemocoel sporozoites intravenously into susceptible animals. Similar to WT parasites, *s6(−)* sporozoites induced patency in a dose-dependent manner *in vivo* (Table 1). This finding confirms that the observed defects in sporozoite locomotion and liver stage development *in vitro* are compensated for *in vivo*. Most importantly, reduced infectivity to the mammalian host is a consequence of the reduced capacity to invade the mosquito salivary glands.

## Discussion

In this study we characterized a member of the parasite motor machinery that plays an important role for sporozoite gliding motility and entry into mosquito salivary glands. Depletion of *S6* by a reverse genetics approach resulted in a consistent reduction of transmission of the malaria parasite to the mammalian host. We could show that the observed decrease in infectivity is a direct consequence of a specific reduction in the loads of mature sporozoites in the salivary glands. This defect in salivary gland invasion correlates with a striking phenotype in *in vitro* gliding motility. Thus, the most important cellular function of *S6* is in mediating sporozoite accumulation in the final target organ of the mosquito vector through its role in sporozoite gliding locomotion.

*Plasmodium*, like other apicomplexan parasites, forms motile extracellular stages that actively penetrate biological barriers and enter target cells. These activities are driven by the parasite's own motor machinery. Central components are transmembrane and surface proteins that link the outside world to the parasite's actin/myosin motor. The founding member of the parasite family of invasins, TRAP, is vital for sporozoite motility and cell invasion (Sultan *et al*., 1997) and appears to function throughout the parasite's journey from the oocysts to the mammalian liver (Sultan *et al*., 1997; Kappe *et al*., 1999; Wengelnik *et al*., 1999; Matuschewski *et al*., 2002a). The parasite apparently expresses tailor-made TRAP/MIC2 family invasins for different life cycle stages. For instance, ookinetes express the invasin circumsporozoite/TRAP-related protein (CTRP) that is essential for ookinete motility and midgut penetration (Dessens *et al*., 1999; Templeton *et al*., 2000) and can be functionally grouped into the TRAP/MIC2 family (Heiss *et al*., 2008). Sporozoites are arguably the most versatile extracellular parasite stages that need to breach numerous barriers along their journey (Amino *et al*., 2006; Baer *et al*., 2007; reviewed in Frevert *et al*., 2008). Not surprisingly, sporozoites express additional TRAP/MIC2 family proteins that together permit efficient life cycle progression. One member, TRAP-like protein (TLP), contains two extacellular adhesion domains, the TSR and the A-domain, yet plays a redundant role in all aspects of sporozoite biology (Heiss *et al*., 2008; Moreira *et al*., 2008). In marked contrast, *s6(−)* mutants display pronounced defects in sporozoite gliding motility and salivary gland invasion. Therefore, *S6* functions appear to closely resemble those of *TRAP* (Sultan *et al*., 1997).

However, two important features differ fundamentally between the two proteins. (i) While TRAP contains classical adhesion modules, namely an A-domain and a TSR, in its extracellular portion, the extracellular region of S6 appears to be lacking any typical adhesion modules. (ii) The observed defects in the *s6(−)* mutants are most prominent in the mosquito phase of the *Plasmodium* life cycle. *trap*(*–*) mutants, in contrast, fail to infect the mammalian host *in vitro* and *in vivo*. Most strikingly, the two proteins do not have redundant but rather distinct roles. Therefore, parasite motility and host cell entry are driven by an array of extracellular proteins that each mediate individual steps from initial substrate and cell recognition to target cell penetration. *S6(−)* parasites largely lost their capacity to glide productively *in vitro* and to accumulate inside salivary glands.

Our findings show that active parasite locomotion, as displayed by haemocoel sporozoites, is necessary for salivary gland entry. In addition to the general sporozoite invasin *TRAP* (Sultan *et al*., 1997) the malaria parasite employs two stage-specific genes, *MAEBL* and *S6*, that function in salivary gland adhesion (Kariu *et al*., 2002) and sporozoite motility respectively. Most importantly, we identified by experimental genetics S6 as the second sporozoite-specific *Plasmodium* protein that has an important role in transducing the motor force from the parasite interior to the extracellular substrate.

## Experimental procedures

### *P. berghei* life cycle

*Anopheles stephensi* mosquitoes were raised at 28°C, 75% humidity, under a 12 h light/dark cycle, and maintained on a 10% sucrose solution during adult stages. For infections with clonal *P. berghei* parasites (ANKA strain, GFP-507cl; Janse *et al*., 2006), 4-day-old female mosquitoes were fed on anaesthetized NMRI mice, which had been infected with *P. berghei* WT parasites or the isogenic *s6(−)* parasites. Parasitaemia was determined for the presence of gametocyte-stage parasites capable of exflagellation. After infection the mosquitoes were maintained at 20°C and 80% humidity. Dissections were performed at days 10, and 14–18, to determine infectivity, and perform a detailed spatial and temporal analysis of the sporozoite populations. Oocyst, haemocoel and salivary gland-associated sporozoites were separated and analysed as described (Vanderberg, 1975).

### *S6* expression analysis

Detection of *S6* transcripts was performed by semi-quantitative RT-PCR. A total of 5 × 10^5^ WT sporozoites were collected from oocysts, haemocoel or salivary glands, and poly (A^+^) RNA was isolated using oligo dT-columns (Invitrogen). cDNA synthesis was performed after DNase I digestion (Ambion) with random decamer primers (Ambion). *S6* transcript abundance was determined using primers S6RTfor (5′-GTGTTATCAACCTTCATATTATTATC-3′) and S6RTrev (5′-CTCCACTTTCGAAAAAATATACAG-3′) and *CSP* primers CSPfor (5′-GACGATTCTTATATCCCAAGCGC-3′) and CSPrev (5′-CCTAATGAATTGCTTACAATATTAAATATACTTG-3′) for normalization.

### *PbS6* gene targeting

For targeted deletion of *PbS6* the gene replacement approach was followed (Thathy and Ménard, 2002). Two targeting fragments were selected from the non-coding regions of *S6* and amplified with *P. berghei* genomic DNA as a template using the following primers: (i) S6rep1Ifor (5′-TCCCCGCGGGCACTTAATATATGCGATTATGGG-3′; SacII site is underlined) and S6repIIrev (5′-CGGGATCCTTTACTCGGTTGTCTATGAATGC-3′; BamHI site is underlined) for a 1045 bp fragment of the 5′ UTR; (ii) S6rep3for (5′-CCCCAAGCTTTATAGACATGGAACACA AAGAGGATAGC-3′; HindIII site is underlined) and S6rep4rev (5′-GGGGTACCTTCTACGAAATCATCTAGTATGCC-3′; KpnI site is underlined) for a 807 bp fragment of the 3′ UTR. Cloning of the two fragments into the *P. berghei* targeting vector flanking the *Tgdhfr/ts*-positive selection marker that provides resistance to the antifolate pyrimethamine resulted in the plasmid pMS01. The targeting vector was linearized with KpnI/SacII and parasite transfection, positive selection and parasite cloning were performed with the fluorescent *P. berghei* ANKA strain as described (Janse *et al*., 2006). Three independent *s6(−)* clonal parasite populations were obtained and tested for *Plasmodium* life cycle progression. The detailed phenotypical analysis was performed with one representative clone. Replacement-specific PCR amplifications of the *s6(−)* locus was performed with specific primer pairs that amplify either the WT or the mutant gene loci.

### Western blotting and immunofluorescence

To generate polyclonal antisera a recombinant amino-terminally His-tagged S6 polypeptide encompassing 156 central amino acid residues, selected for favourable antigenicity, was expressed in an *Escherichia coli* expression vector in BL21 (DE3) cells. The purified recombinant protein was used to raise polyclonal antibodies in pre-screened rabbits housed in a SPF facility (Eurogentec, Seraing, Belgium). For Western blot analysis 10^5^ sporozoites of the different developmental stages were homogenized in SDS sample buffer and boiled. Proteins were separated on 6% polyacrylamide gels and transferred to nitrocellulose membranes by electroblotting. Membranes were blocked, and incubated with the S6 polyclonal antisera (1:1000) or the monoclonal anti-CSP antibody (1:10 000; Potocnjak *et al*., 1980). Bound antibodies were detected using horseradish peroxidase-conjugated anti-rabbit and anti-mouse antibodies respectively (Sigma-Aldrich).

For sporozoite immunostaining and gliding motility assays haemocoel sporozoites of WT and *s6(−)* parasites were isolated by gentle perfusion in RPMI/3% bovine serum albumin (BSA) at days 15–16 post feeding. Pooled haemocoel sporozoites were transferred to BSA-precoated glass coverslips, incubated for 15 min at 37°C in a humid chamber, and fixed in 4% formaldehyde. For membrane extraction, parasites were treated with 1% Triton X-100 (Sigma) in PBS prior to fixation (Bergman *et al*., 2003). For permeabilization sporozoites were incubated with 0.05% Saponin (Sigma) in PBS/1% FCS. Non-permeabilized and permeabilized sporozoites were incubated with the S6 polyclonal antisera (1:100) or the monoclonal anti-CSP antibody (1:1000). Bound antibodies were detected with Alexa-Fluor 546-conjugated anti-rabbit IgG and Alexa-Fluor 488-conjugated anti-mouse IgG respectively (Molecular Probes).

### Analysis of sporozoite infectivity

To determine sporozoite infectivity *in vivo*, *s6(−)* and WT haemocoel sporozoites were injected intravenously at the numbers indicated into young Sprague/Dawley rats. Patency was checked daily by Giemsa-stained blood smears for at least 14 days. The prepatent period is defined as the time until occurrence of the first blood-stage parasites.

For determination of sporozoite infectivity *in vitro*, haemocoel sporozoites were added to subconfluent monolayers of HuH7 cells at the numbers indicated, incubated for 90 min at 37°C and washed off. Liver stages were revealed after 48 h using primary antibodies against *P. berghei* HSP 70 (Tsuji *et al*., 1994) and Alexa-Fluor 488-conjugated anti-mouse IgG (Molecular Probes). To score sporozoite invasion a two-colour assay was performed as described previously (Rénia *et al*., 1988). Briefly, HuH7 cells were grown to subconfluency, incubated for 90 min with the haemocoel sporozoite suspension and washed in DMEM medium containing 10% FCS. To distinguish between extra- and intracellular parasites, extracellular parasites were labelled with the monoclonal anti-CSP antibody, followed by Alexa-Fluor 546-conjugated anti-mouse IgG (Molecular Probes). Permeabilization was performed with ice-cold methanol and re-labelling with the monoclonal anti-CSP antibody, followed by Alexa-Fluor 488-conjugated anti-mouse IgG (Molecular Probes).

### Nucleotide sequence accession number

The nucleotide sequence reported in this article has been submitted to the GenBank database with the Accession No. FJ160771.

## Supporting information

**Movie S1.** Non-productive gliding motility of *s6(−)* haemocoel sporozoites on glass slides, co-incubated with 3% bovine serum albumine. Total time: 20 sec.
